# Impact of a structured yoga program on blood pressure reduction among hypertensive patients: study protocol for a pragmatic randomized multicenter trial in primary health care settings in Nepal

**DOI:** 10.1186/s12906-018-2275-9

**Published:** 2018-07-05

**Authors:** Raja Ram Dhungana, Mahesh Kumar Khanal, Suira Joshi, Om Prakash Kalauni, Anu Shakya, Vijay Bhrutel, Savyata Panthi, Ramesh Kumar KC, Binod Ghimire, Achyut Raj Pandey, Bihungum Bista, Binaya Sapkota, Shiva Ram Khatiwoda, Craig Steven McLachlan, Dinesh Neupane

**Affiliations:** 1 Nepal Family Development Foundation, Kathmandu, Nepal; 2 Ministry of Health, Kathmandu, Nepal; 3 Nepal Ayurveda Research and Training Center, Kathmandu, Nepal; 4 Nepal Health Research Council, Kathmandu, Nepal; 5 Nobel College, Kathmandu, Nepal; 6 Patanjali Ayurveda Medical College and Research Center, Dulikhel, Kathmandu Nepal; 7 Rural Clinical School, University of New South Wales, Sydney, Australia; 8 Nepal Development Society, Bharatpur, Chitwan 10 Nepal

**Keywords:** Hypertension, Obesity, Primary health care, Yoga, Nepal

## Abstract

**Background:**

Hypertension control remains a major global challenge. The behavioral approaches recommended for blood pressure reduction are stress reduction, increased exercise and healthy dietary habits. Some study findings suggest that yoga has a beneficial effect in reducing blood pressure. However, the role of yoga on blood pressure has received little attention in existing health care practices in developing countries. This study will be conducted in primary health care facilities in Nepal to assess the effectiveness of a pragmatic yoga intervention to complement standard practice in further reducing blood pressure.

**Methods:**

This will be multicentric, two arms, randomized, nonblinded, pragmatic trial. It will be conducted in seven District Ayurveda Health Centers (DAHCs) in Nepal between July 2017 and June 2018. The study participants will consist of hypertensive patients with or without antihypertensive medication attending to the outpatient department (OPD). One hundred and forty participants will be randomized to treatment or control groups by using a stratified block randomization. At the study site, the treatment arm participants will receive an intervention consisting of five days of structured yoga training and practice of the same package at home with a recommendation of five days a week for the following 90 days. Both the intervention and control groups will receive two hours of health education on lifestyle modifications. The primary outcome of this trial will be the change in systolic blood pressure and it will be assessed after 90 days of the intervention.

**Discussion:**

This study will establish the extent to which a yoga intervention package can help reduce blood pressure in hypertensive patients. If proven effective, study findings may be used to recommend the governing bodies and other stakeholders for the integration of yoga in the national healthcare system for the treatment and control of hypertension.

**Trial registration:**

Clinical Trial Registry- India (CTRI); CTRI Reg. No- CTRI/2017/02/007822. Registered on 10/02/2017.

## Background

Hypertension is a major public health issue worldwide. Globally, 874 million adults had systolic blood pressure (SBP) of 140 mmHg or higher in 2015 [1]. Recent evidence suggested that nearly one third of Nepali had high blood pressure [2]. Hypertension is one of the established risk factors for stroke, myocardial infarction, congestive heart failure, arterial aneurysm and chronic renal failure [3]. Risk of cardiovascular disease multiplies for each 20/10 mmHg increment of systolic/diastolic blood pressure above normal blood pressure cut-offs [4].

Prevention and management of hypertension is a global public health challenge. Apart from antihypertensive medicines, lifestyle modifications have been recommended as an equal first line approach for controlling hypertension [4, 5]. Healthy lifestyle such as weight loss, dietary sodium and total fat reduction, limiting alcohol intake, increased intake of vegetables and fruit, quitting smoking and regular physical exercise may lower SBP in the range of 4–9 mmHg [5, 6].

Use of complementary and alternative medicine (CAM) for treating high blood pressure is becoming increasingly popular [7]. A trial conducted in Thailand demonstrated that yoga treatment was associated with significant reduction in blood pressure compared with the control group (SBP: mean difference − 26.74 mmHg; diastolic blood pressure (DBP): − 19.80 mmHg) [8]. Deepa et al. found a significant fall in mean blood pressure after three months of yoga practice (*p* < 0.00001) (SBP: -9.92 mmHg; DBP: − 9.83 mmHg) among the mild to moderate essential hypertensives [9]. Similarly, a study conducted in eastern Nepal reported that yoga significantly reduced the blood pressure level (SBP: 144 mmHg Vs 130 mmHg; *p* = 0.018, and DBP: 98 mmHg Vs 88 mmHg; p = 0.018) among the essential hypertensive participants [10]. In a Cochrane review conducted by Hartley L et al., yoga was found to significantly lower DBP (Mean deviation − 2.9 mmHg, 95% CI -4.52 mmHg to − 1.28 mmHg) [11]. However, out of 11 studies analyzed, six studies confirmed that there was no significant reduction in SBP and one study, in contrast, reported an increase (Mean deviation 2.2 mmHg) in SBP [11]. A definite conclusion over the therapeutic use of yoga as an adjunct to hypertension therapy is lacking because of inconsistency in findings as well as non-uniformity in yoga prescription [12–14]. Moreover, these systematic reviews have recommended for larger and methodologically sound studies to determine the impact of yoga on hypertension [12–14].

This study aims to assess the effectiveness of structured yoga program in reducing blood pressure level in hypertensive patients attending DAHCs in Nepal. The study will be conducted in real-world clinical practice settings, with typical patients and by government health workers, who are not the professional yoga instructors. If the study finding is significant, yoga may be integrated as a complementary therapy for hypertension management in public health facilities in Nepal.

## Primary objectives

The primary objective of the study is to assess the effect of structured yoga practice on blood pressure reduction among people with hypertension attending the primary healthcare facilities. This study will determine whether practicing yoga leads to a clinically relevant reduction in blood pressure. Both clinic management and follow-on home-based yoga practice among hypertensive participants will be in the prescribed program. The outcome will be the change in systolic blood pressure at 90 days. We will also record the change in diastolic blood pressure. The secondary aim of the study is to assess the effect of yoga in reducing body mass index among the same participants.

## Methods

### Trial design

This study will be conducted among 140 hypertensive participants in seven District Ayurveda Health Centers in Nepal. It will be a randomized, two-armed, non-blinded, pragmatic trial.

### Study settings

This study will be conducted in seven purposively selected DAHCs in Nepal. Currently, two centrals, 14 Zonal and 61 District Ayurveda Health Centers are catering preventive, promotive & curative primary healthcare services throughout Nepal under Department of Ayurveda (DoA), Ministry of Health, Nepal. Recently, DoA has initiated Training of Trainers (ToT) of yoga to capacitate its technical staffs (Ayurveda doctors and paramedics working at DAHC) to educate the patients and community on basic yoga skill. The main objective of ToT is to integrate and promote yoga within Ayurveda health system for health promotion and disease prevention. This study will orient and mobilize the trained DAHC human resources to deliver yoga interventions for the hypertensive participants those attending primary healthcare centers.

**Fig. 1:**
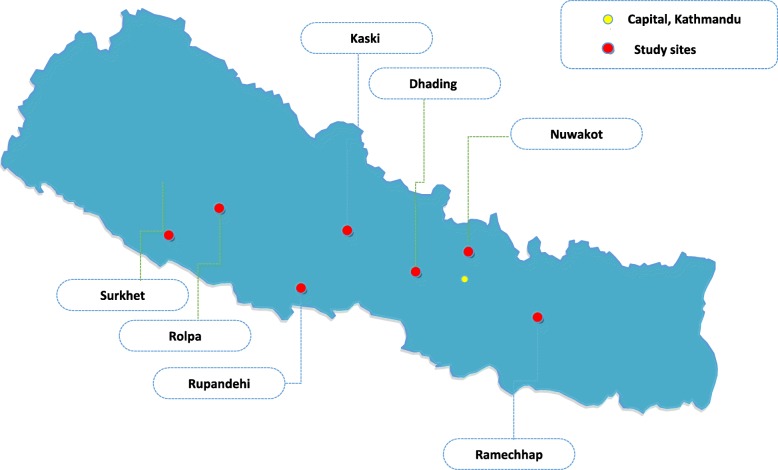
Location of study sites. Seven Ayurveda Health Centers are selected from Ramechhap, Dhading, Nuwakot, Kaski, Rupandehi, Surkhet and Rolpa Districts

This study has purposively selected seven DAHCs from the following districts: Ramechhap, Dhading, Nuwakot, Kaski, Rupandehi, Surkhet and Rolpa- based on ecological belt and development region (Fig. 1).

### Study participants

Study participants will include hypertensive patients with or without anti-hypertensive medications attending DAHCs. Inclusion criteria include a SBP between 130 and 159 mmHg and/or DBP between 85 and 99 mmHg. Following are the sample selection criteria in detail.

#### Inclusion criteria

- Age between18 years and 70 years
- BP criteria: SBP of 130 to 159 mmHg or DBP of 85 to 99 mmHg
- 3 months or more of prescribed antihypertensive medication or without previously prescribed anti-hypertensive medications
- Attending to DAHCs

#### Exclusion criteria

- Person having diabetes (self-reported) and mental diseases
- Pregnant women
- Known secondary cause of hypertension (self-reported)
- Hypertension (SBP ≥160 mmHg or DBP ≥100 mmHg)
- Practicing yoga more than one month in the previous 6 months

### Intervention

The intervention consists of three components: a) five days of yoga training (two hours per day); b) two hours health education session on healthy lifestyles; and c) home based self-practice of yoga daily for 90 days (with daily session lasting for 30 min). The treatment arm participants will receive all the intervention components whereas the control group will only be given the two-hour health education program and standard clinic care. Before the intervention, District Ayurveda Health Center staff (one from each center) will be orientated on yoga intervention packages and health education for high blood pressure prevention.

Structured yoga program contains yoga postures, breathing exercise and meditation (Table 1). Studies have used different regimens and elements of yoga intervention such as warm-up exercise [15, 16], postures [17–20], breathing [15, 18–20], Om recitation [16, 17, 20], relaxation [20, 21] and meditation [17, 18, 21] in single or combination generally for 30 min per day [15] for three months [9]. Hagins et al., in their systematic review suggested to integrate three elements of practice (posture, meditation, and breathing) as a single component. This combination of yoga practice was associated with significant reductions in blood pressure whereas yoga interventions using two or fewer elements of yoga practice or that combined yoga practice with additional interventions were not [14]. The intervention package in the current study has been developed based upon the recommendation from yoga therapists located in yoga centers in Nepal and aforementioned published studies [14]. Each yoga session takes 30 min to complete (Table 1). It starts with Om recitation (1 min), followed by warm up exercises (5 min), postures (4 min), relaxation (3 min), breathing (8 min) and meditation (9 min). One staff from each center will be trained in the yoga intervention package. The same person will deliver the yoga intervention to the high blood pressure participants. The study participants will receive a yoga training for two hours over five days at the allocated DAHCs. After the completion of training, participants in the treatment group will be requested to continue the practice at home once every morning for next 90 days. They will be invited for the regular follow-up to the centers once every 30 days. Participants will be asked to record the number of days they practice yoga using a calendar sheet to monitor progress and encourage accountability. During each follow-up day, outcome measurements (blood pressure and BMI) as well as qualitative assessment of home-based practice will be assessed.

**Table 1:** Yoga module for the study participants

Sn	Practices	Rounds	Duration
1.	Starting with Omkar (Om recitation)	3	1 min
2.	Loosening exercise with synchronization of breathing in sitting position a) Toe bending b) Ankle bending c) Knee bending d) Half butterfly e) Finger bending f) Wrist bending g) Elbow bending h) Shoulder rotation i) Neck bending up and down	1 for each	5 min
3.	Ardhakatichakrasan (Lateral Arc Pose)		2 min
4.	Vakrasana (Twist Pose)		2 min
5.	Yogic abdominal awareness, breathing and feeling in Shavasana		3 min
6.	Chandravedi Pranayam (Left nostril breathing)	9 rounds	2 min
7.	Sheetali Pranayam (Cooling breathing)	9 rounds	2 min
8.	Nadi Suddhi Pranayama (Alternate Nostril breathing)	9 rounds	2 min
9.	Bhramari (Humming bee breathing**)**	9 rounds	2 min
10.	Yoga nidra (Yogic sleep)		9 min
	Total Time		30 min

Health education group counseling session (a single 2-h workshop) will be organized for both intervention and control groups. The course curriculum and contents have been adopted from the health education materials endorsed by National Health Education Information and Communication Center (NHEICC), Ministry of Health, Nepal. It contains behavioral and lifestyle modification education designed to reduce an exposure to the risk factors of hypertension like alcohol consumption, smoking, physical inactivity, and fat and salt reduction in diet (Table 2).

**Table 2:** Health education module for the study participants

Sn	Chapter	Contents	Method	Media/tools	Time (Min)
1	Assessing level of understanding about hypertension among participants	Assessing paticipants’ knowledge on hypertension and its risk factors, complications and management	Brainstorming		15
2	A brief introduction on high blood pressure	Understanding the meaning of high blood pressure and its burden in Nepal	Interactive lecture	Drawing paper Marker	10
3	Risk factors of high blood pressure	Causes, non-modifiable risk factors, modifiable risk factors	Interactive lecture	Drawing paper Pictures, cartoon, Marker	10
4	Complication of high blood pressure	Complications of high blood pressure	Interactive lecture	Drawing paper Picture showing complication, Marker	10
	Break				15
5	Behavioral approaches for the management of high blood pressure	Healthy eating, nutritional recommendation based on Dietary approaches to stop hypertension (DASH), physical activity, maintaining healthy weight, limiting (or avoid) alcohol, quitting tobacco, reducing stress, tips for changing daily life	Interactive lecture, sharing of success case study	Drawing paper Pictures, Marker	60

### Outcomes

The primary outcome of this trial will be the change in systolic blood pressure. It will be measured before, on each month interval and after completion of the intervention (at 90 days) at outpatient clinical settings with an aneroid sphygmomanometer (BP set). The secondary outcome will be the body mass index (BMI) and heart rate (HR). Both will be assessed at baseline and after the intervention.

### Sample size

Sample size calculations are based on continuous outcome superiority trial; assuming 7 mmHg of mean difference in systolic blood pressure between control and experimental groups [14]. This study requires 140 participants to have a 80% chance of detecting the proposed change in systolic blood pressure in the experimental group as significant at the 5% level, after adjusting 20% dropout rate [22]. A standard deviation of outcome variable was estimated as 13 mmHg in both arms [23]. As recommended by the literature, design effect of this multicenter study is considered very negligible and excluded from the calculation [24].

### Recruitments

**Fig. 2:**
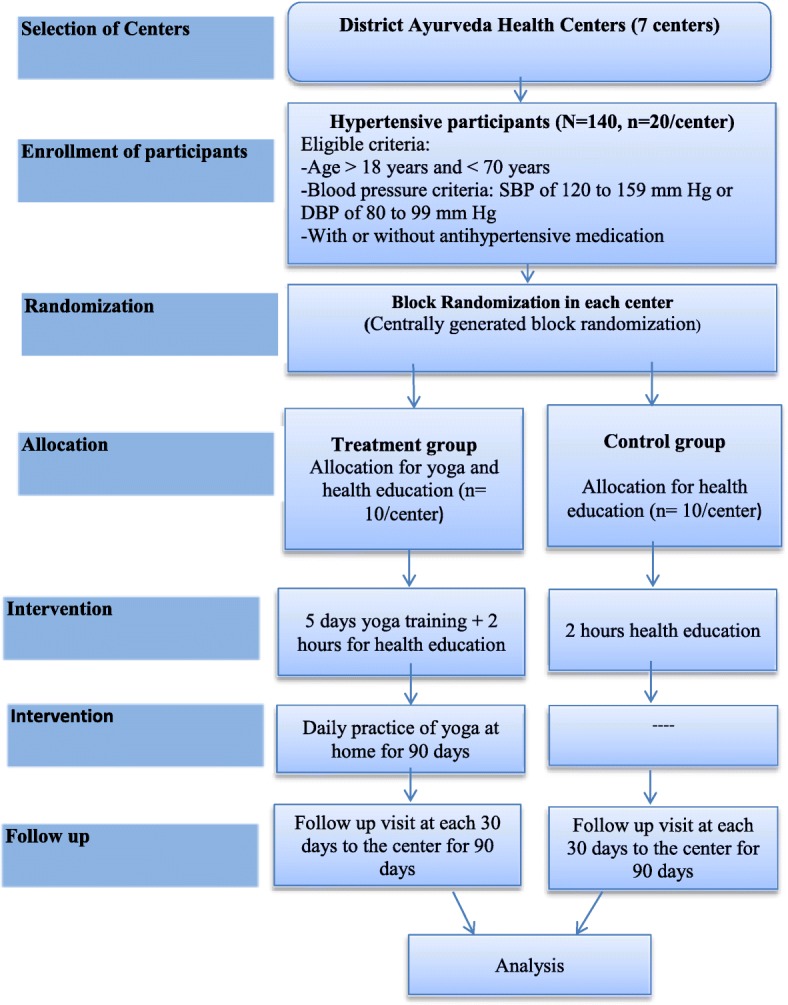
Flow chart of phases of study. Total 140 study participants from seven study sites (20 per site) will be enrolled in the study. Both arms (treatment and control) will contain equal numbers of participants (10 in each arm) at each center (through stratified block randomization)

Both the DAHC research staff and clinicians will be trained on the protocols and procedures of the study. They will screen the hypertensive patients attending DAHCs for the eligibility criteria; obtain written consent; enroll the eligible participants for the study; and send the participants’ name for centrally generated stratified blocks for randomization (Fig. 2). That is to allocate the participants in treatment and control arm. The randomization scheme will consist of a sequence of blocks such that each block will contain a pre-specified number of treatment assignments in random order. For this, out of total 20 subjects in each center, 4 blocks of various sizes will be randomly created and participants will be allocated in two-arms achieving balance across groups (10 in treatment; 10 in control). Participant list will be prepared by someone not involved in the recruitment to the trial and will be shared each center.

### Data collection

Baseline and post-intervention data on primary and secondary outcomes will be collected during a face to face interview. Additionally, anthropometric measurements and clinical examination data will also be collected. This study will also collect pre-intervention information on tobacco use, alcohol consumption, dietary patterns and physical activity.

#### Face-to-face interview

The interview questionnaire will cover participant’s demographics and health behavior. The health behavior questions are designed to assess tobacco use, alcohol consumption, fruit and vegetable consumption and physical activity. Tobacco, alcohol and diet related questions will obtain information on frequency and pattern of use or intake. These questions are particularly adopted from STEPs survey questionnaire tool [25]. For measuring physical activity level, seven days history of physical activity will be recorded and converted into Metabolic Equivalent of Task (MET) value using available guidelines [26].

#### Anthropometric measurement

To assess the change in BMI, height and weight will be measured using portable stadiometer and digital scales [27].

#### Clinical measurement

Blood pressure will be measured with an aneroid sphygmomanometer (BP set) in outpatient settings. Before taking the measurements, participants will be requested to rest for 15 min with legs uncrossed. Three readings of the systolic and diastolic blood pressure will be recorded in each five-minute interval. The average of second and third readings will be used for analysis.

### Data management and analysis

Data will be complied, edited, processed and analyzed using Epidata 3.1 and Stata 14.0. Analyses will be performed based on the intention-to-treat (ITT) principle. In the case of missing data and lost to follow-up, the last data obtained from a participant will be used for analysis. Similarly, for the participants who will start new medication or change the dose of existing medication during the study period, data will be replaced via the last observation carried forward method. Sensitivity analyses will be performed to test whether the findings from the available-case analysis are consistent and will lead to similar conclusions as in the primary analysis. A per-protocol (PP) analysis will also be carried out for only those patients who will complete the treatment as allocated.

Baseline characteristics will be presented in tables with frequency, proportions and mean ± standard deviation; frequency comparisons will be made using Chi-squared tests, and independent sample t-tests for mean differences. Primary and secondary outcomes will be analyzed with mixed-effects linear regression models where each health center will allow to have a random intercept. Potential confounding variables including smoking, alcohol consumption and physical activity will be treated as fixed factors. Both models will include baseline assessments as covariates. All tests will be two-tailed and *p* < 0.05 will be considered statistically significant.

### Data and safety monitoring

To ensure the data safety, a data monitoring and quality assurance team will review the procedures for maintaining the confidentiality of data, and the quality of data collection, management and analyses. They will assess the missing information and errors in the dataset. They will also closely monitor recruitment, participation status, treatment compliance, regular follow-up and overall quality of research, and provide necessary feedback to the study team.

The central and district research team will review participants’ data regularly to ensure safety of the trial. Research Investigators, mostly DAHC clinicians, located in each district will be responsible for reporting serious adverse events and unanticipated problems to the Principal Investigator, who will inform Ethical Review Board (ERB) within 48 h of the events. The district research team will also quickly follow up, assess and treat or refer the participants as required.

## Discussion

Hypertension is emerging as one of the major public health concerns in Nepal. Risk factors survey of the non-communicable disease (NCD) reported that 25.7% of all study population had hypertension [28]. Similarly, large proportions of hypertensive participants often have uncontrolled blood pressure [29].

Global action plan for the prevention and control of non-communicable diseases envisages for a 25% relative reduction in the prevalence of raised blood pressure in the next 10 years. Nepal has a well formed contextualized plan [30]. Interestingly, yoga as a health promotion measure has been recently established via the Urban Health Promotion Centers. Similarly, Department of Ayurveda introduced five days of yoga training to its sub-centers’ staffs for integrating yoga in prevention and control of NCDs. However, we still do not have sufficient evidence on the effect of yoga on hypertension prevention and control in Nepalese community. Therefore, this study expects to generate evidence that will be widely applicable for assisting blood pressure control and well-being in clinical and community settings in Nepal. It will be conducted in existing primary healthcare settings where the intervention package will be delivered by the government staffs at their respective OPDs. Such real-life routine practice settings for intervention would possibly increase the likelihood of generability and applicability of the study findings [31]. If proven effective, yoga can be integrated with medication as an adjunctive therapy for blood pressure control in similar low resource settings of Nepal and developing countries.

This study is not designed to determine which components of the yoga intervention will have an effect on blood pressure. To minimize errors in blood pressure measurement, data enumerators (medical persons) will be uniformly trained in blood pressure measurement including proper timing and positioning of the cuff and the patient.

## Abbreviations

BMI: Body Mass Index
BP: Blood Pressure
CAM: Complementary and alternative medicine
CTRI: Clinical Trial Registry- India
CVD: Cardiovascular Disease
DAHC: District Ayurveda Health Center
DBP: Diastolic Blood Pressure
DoA: Department of Ayurveda
ERB: Ethical Review Board
HR: Heart rate
ITT: Intention to treat
MET: Metabolic Equivalent
NCD: Non-communicable disease
NHEICC: National Health Education Information and Communication Center
OPD: Outpatient department
PP: Per protocol
SBP: Systolic Blood Pressure
ToT: Training of Trainers
